# Effects of *Lentilactobacillus buchneri* and *Kazachstania bulderi* on the Quality and Flavor of Guizhou Fermented Red Sour Soup

**DOI:** 10.3390/foods12203753

**Published:** 2023-10-12

**Authors:** Na Liu, Xiuli Li, Yue Hu, Likang Qin, Aiming Bao, Weijun Qin, Song Miao

**Affiliations:** 1 Key Laboratory of Plant Resource Conservation and Germplasm Innovation in Mountainous Region (Ministry of Education), School of Liquor and Food Engineering, Guizhou University, Guiyang 550025, China; naliu@gzu.edu.cn (N.L.); 15772235609@139.com (X.L.); huyue2015@163.com (Y.H.); 2 Teagasc Food Research Centre, Moorepark, Fermoy, P61 C996 Cork, Ireland; 3 Guizhou Nanshanpo Food Processing Co., Ltd., Anshun 561000, China; aiming.bao@nanshanpogroup.com (A.B.); weijun.qin@nanshanpogroup.com (W.Q.)

**Keywords:** red sour soup, fortified fermentation, *Lentilactobacillus buchneri*, *Kazachstania bulderi*, flavor and quality

## Abstract

In this study, the effects of *Lentilactobacillus buchneri* (*L. buchneri*: CCTCC M 2023228) and *Kazachstania bulderi* (*K. bulderi*: CCTCC M 2023227) on the quality characteristics and volatile flavor substances in fermented red sour soup were explored based on natural fermentation. Compared to natural fermentation (nitrite: 5.5 mg/kg; amino acid nitrogen: 0.17 g/100 g; lycopene: 63.73 µg/mL), three fortified fermentation methods using *L. buchneri*, *K. bulderi*, and both strains together significantly reduced the concentrations of nitrite (2.62, 2.49, and 2.37 mg/kg), amino acid nitrogen (0.03 g/100 g, 0.02 g/100 g, and 0.05 g/100 g), and lycopene (26.64, 32.45, and 51.89 µg/mL). Total acid content (11.53 g/kg) and lactic acid bacteria count (285.9 ± 1.65 × 10^6^ CFU/mL) were the elements most significantly increased by fortified fermentation with *L. buchneri* relative to other fermentation methods. A total of 99 volatile compounds were determined in red sour soup and could be roughly classified into alcohols, aldehydes, ketones, and esters. Fortified fermentation with two strains and fortified fermentation with *K. bulderi* increased the content of methyl butanoate and 3-hydroxybutan-2-one-acetoin (D). This study confirmed the effects of *L. buchneri* and *K. bulderi* on the quality and flavor of fermented red sour soup and provided a theoretical basis for the fortified fermentation of red sour soup.

## 1. Introduction

Red sour soup is a condiment made from tomatoes and red peppers and supplemented with some ginger, salt, and white wine [1]. It originates from the southwestern region of Guizhou, China, and has a history of more than one thousand years. Fermented red sour soup contains many organic acids, minerals, amino acids, capsaicin, and lycopene [2] and plays an important role in preventing nonalcoholic fatty liver disease, regulating intestinal flora, scavenging free radicals, enhancing immunity, and maintaining the acid–base balance [3,4,5]. Currently, red sour soup has become an essential food for daily cooking in southwest China [1] and is widely used in the production of hot pot seasonings and sour fish products.

Flavor, color, and aroma are three key quality indicators of foods [6]. Volatile aroma compounds significantly affect the flavor and overall evaluation of foods [7]. Studies have revealed that the flavor of fermented foods is closely related to microbial communities [8,9,10,11]. Fermenters affect the diversity and structure of microbial communities during fermentation [12]. The fermentation agents of fermented foods have been explored. Liu et al. [13] used a mixture of *L. paracasei* and *K. marxianus* to increase the acidity and aroma of rice sour soup and shorten its fermentation time. Song et al. [14] reported that inoculation with *L. plantarum* and *P. pentosaceus* increased the content of amino acids, alcohols, and aldehydes in sauerkraut. In Zheng et al. [15] and our previous studies [16], we found that the total acid content of the fortified fermented red sour soup reached the maximum level and was higher than the T/TSSP standard for fermented fruit and vegetable juices (pulps) and their products on the fifth and sixth days [17]. Fermentation agents play an important role in improving food quality. However, fortified fermentation with the dominant strains from red sour soup has seldom been explored.

Lactic acid bacteria (LAB) and yeasts are the most commonly used microorganisms in food fermentation. Niamahr et al. [18] used LAB and yeasts to ferment yogurt and improved the physical and chemical properties of the yogurt. Zhong et al. [19] found that the co-fermentation of LAB and non-saccharomyces cerevisiae affected the antioxidant properties, aroma compounds, and total acids of mango juice. Various LAB and yeasts can increase the lactic acidity, ester aroma, and mellow aroma of fermented products [20]. Lactobacillus and yeasts are the main strains in the fermentation of red sour soup [1] and largely affect its flavor and quality. To date, *L. plantarum NR1-7*, *Bifidobacteriµm animalis* subsp. *lactis BZ11*, and *Candida utilis RY* have been used as fermenters in red sour soup to shorten the fermentation time of red sour soup and increase the content of lycopene, 6-gingerin, capsaicin, and lactic acid [21].

Gas chromatography–ion-mobility spectrometry (GC-IMS) is a powerful technique for the separation and sensitive detection of volatile organic compounds and has the characteristics of fast response speed, high sensitivity, convenient operation, and low cost [7]. It has a wide range of applications in foods and can be used to determine adulterated foods, stale foods, and volatile metabolites in the process of food processing [22,23,24].

The traditional fermentation process of red sour soup is affected by raw materials, microbial community, fermentation time, and fermentation temperature and the fortified fermentation of red sour soup has seldom been reported. Fermentation with the dominant bacteria screened from red sour soup may help to improve its quality and flavor. Therefore, we investigated the effects of dominant LAB and/or yeasts on the physicochemical properties, organic acids, lycopene, and volatile flavor components of red sour soup. This study provided a theoretical basis for the fortified fermentation of red sour soup.

## 2. Materials and Methods

### 2.1. Materials

Ingredients for preparing sour soup, including tomatoes, chillies, garlic, ginger, salt, and glutinous rice, were purchased from Shiban Logistics Park, Huaxi District, Guiyang City, Guizhou Province. *Lentilactobacillus buchneri* (*L. buchneri*: CCTCC M 2023228) and *Kazachstania bulderi* (*K. bulderi*: CCTCC M 2023227) are the strains in red sour soup screened by our group. De Man–Rogosa–Sharpe (MRS), modified Chalmers agar (MC) and yeast extract peptone dextrose medium (YPD agar medium (YPD) and YPED broth medium (YPED)) were purchased from Shanghai Bo Microbiology Technology Co. (Shanghai, China). Lycopene standard, tartaric acid, citric acid, and lactic acid were purchased from Beijing Solaibao Technology Co. (Beijing, China). Formaldehyde and sodium hydroxide were purchased from Xilong Science Co. (Shenzhen, China). Phenolphthalein, zinc acetate, p-aminobenzenesulfonic acid, naphthalene ethylenediamine hydrochloride, and sodium borate were purchased from Shanghai Yien Chemical Technology Co. (Shanghai, China). Sodium chloride, anhydrous ethanol, potassium ferricyanide, etc., were purchased from Tianjin Fuyu Fine Chemical Co. (Tianjin, China).

### 2.2. Strains, Culture Media, and Growth Conditions

*L. buchneri* and *K. bulderi* were screened by our group in Guizhou red sour soup. LAB and yeasts were cultured according to the method of Liu et al. [25] with minor modifications. *L. buchneri* was inoculated in MRS agar at 37 °C for 1 d and purified for 3 generations. Then, the purified *L. buchneri* was inoculated in MRS broth at 37 °C and cultured at 150 RPM for 30 h to obtain the primary seed liquid, which was then inoculated into MRS broth at 3% inoculation rate so that the secondary seed liquid could be obtained under the same conditions. *K. bulderi* was inoculated in YPD at 30 °C for 24 h and purified for three generations. After that, the purified *K. bulderi* was inoculated in YEPD at 30 °C and cultured at 150 RPM for 24 h to obtain primary seed liquid, which was then inoculated into YEPD at 3% inoculation rate so that the secondary seed medium could be obtained under the same conditions.

### 2.3. Preparation of Red Sour Soup Samples

Red sour soup samples were prepared according to a previous report, with minor modifications [26]. Firstly, tomatoes were mixed with other ingredients, including chillies (36%), salt (13%), glutinous rice (6%), ginger (3%), and garlic (2%), to obtain a sour soup mixture. Afterwards, fermentation was carried out with four fermentation agents: fermentation with *L. buchneri* (3%, mass ratio) alone (MLQ), fermentation with *K. bulderi* (5%, mass ratio) alone (MYQ), fermentation with a mixture of *L. buchneri* (3% mass ratio) and *K. bulderi* (5% mass ratio) (MSQ), and natural fermentation (MZ). Fermentation was carried out in triplicate and samples were acquired on days 1 and 5 of fermentation.

### 2.4. Physical, Microbial and Chemical Determination

The pH values of samples were measured with a pH meter (Testo 205, Titisee-Neustadt, Germany) [27]. *Lactobacillus* counting was performed based on the method of Liu et al. [16]. Firstly, the samples were diluted with sterile saline 10 times in series, and two to three suitable dilutions were selected and spread on MC agar plates and MRS agar. The plates were incubated aerobically at 36 ± 1 °C for 72 ± 2 h and anaerobically at 36 ± 1 °C for 72 ± 2 h, respectively. Yeast counting was performed according to GB 4789.15-2016 Counting of Moulds and Yeasts. The samples were coated on Bengal red agar plates after treatment and incubated at 28 ± 1 °C for 5 d. The counting results were recorded to calculate the total number of yeasts. Total acid content and amino nitrogen content were determined according to the acidity meter method in GB 5009.235–2016 Determination of Amino Nitrogen in Foods [22]. Nitrite content was determined according to GB5009.33-2016. Organic acid content was determined based on a previous method [26], with minor modifications, under the following conditions: column temperature of 35 °C, detection wavelength of 210 nm, injection volume of 10 μL, and flow rate of 0.8 mL/min. Lycopene content was determined according to a previous method [28].

### 2.5. GC-IMS Analysis

Flavors collected from different red sour soup samples after different fermentation days were detected with an Agilent 490 gas chromatograph (Agilent Technologies, Palo Alto, CA, USA) equipped with an automatic sampling device and an IMS instrument (Flavor Spec^®^, Analytical Sensor Systems, Dortmund, Germany). Firstly, 1 g of each red sour soup sample was weighed and added to a 20 mL headspace bottle. The samples were incubated for 15 min (80 °C, 500 RPM). After incubation, 200 μL of headspace sample was automatically withdrawn with a syringe and injected into the GC injector with N_2_ as the carrier gas (column temperature of 60 °C and drift tube temperature of 45 °C). The flow rate was set at 2 mL/min for 2 min, then increased to 10 mL/min within 3 min, increased to 100 mL/min within 20 min, and maintained for 5 min.

### 2.6. Calculation of Relative Odor Activity Value (ROAV)

On the basis of relative quantification, the ROAV values of aroma compounds in water were calculated according to a previous method [11]. The equation for each relative odor activity value is provided as follows:

ROAV = *C_i_*/*C_max_* × *T_max_*/*T_i_* × 100, (1)

where *C_i_* denotes the relative content of aroma compounds in the sour soup (%); *T_i_* is the aroma threshold of the compound in water (μg/kg); *C_max_* and *T_max_*, respectively, denote the relative content and aroma threshold of the compound that contributes most to the overall flavor of the sample.

### 2.7. Statistical Analysis

The analytical software used in the experiment was composed of VOCal and three plug-ins. VOCal was used for viewing analytical spectra and the qualitative and quantitative analysis of data. Reporter plug-in was used for the direct comparison of spectral differences between samples. A gallery plot was used for the fingerprint comparison of volatile organic compounds between different samples. The dynamic PCA plug-in was used for dynamic principal component analysis. One-way analysis of variance (ANOVA) was performed using SPSS software (Version 24, SPSS Inc., Chicago, IL, USA) to determine statistical significance and Duncan’s multiple range test was performed to detect significant inter-sample differences (*p* < 0.05). Origin 8.5 (Origin Lab Corp, Hampton (HPT), USA) was used for data plotting. TB tools version 1.082 (Heatmap Illustrator, toolbox for biologists, China) was used to create heat maps.

## 3. Results

### 3.1. Physical and Chemical Indicators

**Figure 1:**
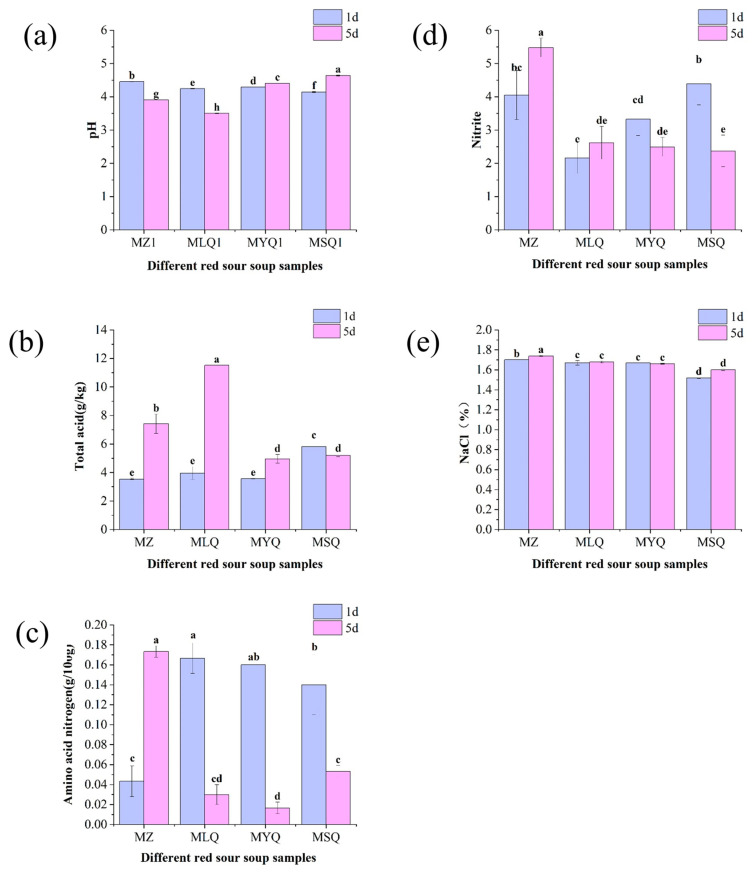
Effect of different fermentation methods on pH (**a**), total acid (**b**), amino acid nitrogen (**c**), nitrite (**d**), and NaCI (**e**) in red sour soup samples. Different small letters indicate significant differences between samples of different red sour soups (*p* < 0.05).

Among all physical indicators, pH is an important parameter indicating the growth status of microorganisms and the accumulation of metabolites, which have an important impact on the quality of fermented foods. The pH values of MZ and MLQ samples decreased from 4.45 ± 0.01 and 4.25 ± 0.01 to 3.91 ± 0.01 and 3.5 ± 0.01, respectively (Figure 1a). After five days of fermentation, the pH values of MYQ and MSQ increased from 4.29 ± 0.01 and 4.14 ± 0.02 to 4.41 ± 0.01 and 4.61 ± 0.02, respectively. LAB can convert sugars into lactic acid and other organic acids [29], thus decreasing the pH value of MLQ samples. Additionally, a lower pH could inhibit the growth of complex microorganisms, thus reducing nitrite levels [30].

Total acid is an important indicator of the sourness of fermented vegetables [31] and mainly related to organic acid metabolism and the degradation of raw proteins and starches by microorganisms. Figure 1b indicates the changes in total acid content in different red sour soup samples from day 1 to day 5 of fermentation. Except for the total acid content of MSQ samples, which decreased, the total acid content of other samples increased. Especially, the total acid content of MLQ samples increased most significantly from 3.56 ± 0.05 g/kg on day 1 to 11.53 ± 0.00 g/kg on day 5. These data met the industrial standard of sour soup (T/TSSP 014–2022), in which the total acid should be more than 5 g/kg (0.5 g/100 mL) [17]. The total acid content was mainly ascribed to the accumulation of organic acids (such as lactic acid) [13].

Amino acid nitrogen affects the umami flavor of fermented vegetables. During the fermentation of red sour soup, proteins were hydrolyzed into various amino acids, which endowed the red sour soup with a good flavor. Amino acid nitrogen content showed significant differences between the various samples (*p* < 0.05, Figure 1c). The amino acid nitrogen content of the MZ sample was 0.04 ± 0.02 c on the first day of fermentation and increased to 0.17 ± 0.01 g/100 g on the fifth day. Natural fermentation had more miscellaneous bacteria and the fermentation was slow, so amino acid nitrogen mostly accumulated on the fifth day. The amino acid nitrogen content of the MLQ, MYQ, and MSQ samples decreased from 0.17 ± 0.02 g/100 g, 0.16 ± 0 g/100 g, and 0.14 ± 0.03 g/100 g to 0.03 ± 0.01 g/100 g, 0.02 ± 0.01 g/100 g, and 0.05 ± 0.01 g/100 g, respectively, because the microbial proliferation in the early stages of fortified fermentation in these samples was intensive and produced higher levels of amino acid nitrogen than natural fermentation.

Nitrite formation and accumulation in fermented or salted vegetables often causes food safety problems [32]. The nitrite content of the MZ and MLQ samples increased from 4.05 ± 0.74 mg/kg and 2.17 ± 0.47 mg/kg to 5.48 ± 0.28 mg/kg and 2.62 ± 0.50 mg/kg, respectively (Figure 1d), because the nitrate reductase secreted by some miscellaneous bacteria on the surface of raw materials reduced the nitrate in raw materials into nitrite. The nitrite content of the MYQ and MSQ samples decreased from 3.33 ± 0.49 mg/kg and 4.39 ± 0.63 mg/kg to 2.50 ± 0.29 mg/kg and 2.37 ± 0.47 mg/kg, respectively, probably due to the effect of yeasts on nitrite reduction. The accumulation of organic acids produced by the inoculated fermentation of LAB significantly reduces the nitrite content of fermented foods [33]. In this study, the inoculation of LAB in fortified fermentation samples had no effect on reducing nitrite due to insufficient fermentation time (Figure 1e). Sodium chloride content showed no significant change from day 1 to day 5 (*p* > 0.05) in all the samples except the MZ sample.

### 3.2. Counting of Microbial Cells

The colony counts of bacteria, LAB, and yeasts in red sour soup samples on day 1 and day 5 of fermentation are shown in Table 1. The total number of bacteria, LAB, and yeast colonies detected in each sample increased significantly. After 5 days of fermentation, the cell densities of bacteria (149.33 ± 4.04 × 10^6^ CFU/mL) and LAB (285.9 ± 1.65 × 10^6^ CFU/mL) in the MSQ sample were higher than those in the other three samples, and the highest cell density of yeasts was found in the MYQ sample on day 5 of fermentation. It was noteworthy that the colony number of bacteria in the MLQ sample on day 5 of fermentation was much larger than that of LAB because the environment of the MLQ1 sample was more suitable for the growth and survival of miscellaneous bacteria. The growth of yeasts was influenced by the LAB fermentation agent. In the MSQ red sour soup sample, the total number of LAB was 5.5 times of that of yeast because of the antagonism between LAB and yeasts under the condition of nutrient competition [34].

### 3.3. Changes in Organic Acids in Different Red Sour Soup Samples

Organic acids are mainly responsible for the flavor of fermented products and their composition and content largely determine the acidity of the final products [35]. Therefore, in this study, the content of seven organic acids (oxalic acid, tartaric acid, malic acid, lactic acid, acetic acid, citric acid, and succinic acid) was examined in different red sour soup samples on day 1 and day 5 of fermentation. Oxalic or tartaric acid was not detected in any sample or only a small content of oxalic or tartaric acid was detected (Table 2). The content of oxalic and tartaric acids showed no significant differences between samples (*p* > 0.05). The content of malic acid did not differ significantly between samples after five days of fermentation. The content of lactic acid in MLQ samples increased significantly from 2.91 ± 0.33 g/kg on day 1 to 13.55 ± 11.77 g/kg on day 5, whereas the content of lactic acid in MSQ samples decreased from 3.37 ± 1.06 g/kg on day 1 to 0.22 ± 0.19 g/kg on day 5, probably due to the interaction between LAB and yeasts. The content of acetic acid in all samples increased during fermentation. Especially, the content of acetic acid in the MSQ sample increased from 1.22 ± 0.77 g/kg to 3.41 ± 0.29 g/kg. The citric acid content of all samples decreased significantly (*p* < 0.05). Citric acid content in the MSQ sample decreased from 4.33 ± 0.06 g/kg to 0 g/kg because the metabolism of citric acid by LAB produced lactic acid, diacetyl, or other substances [36]. Succinic acid was not detected in MZ samples and the content of succinic acid detected in the remaining samples increased on day 5.

### 3.4. Changes in Lycopene Content in Different Red Sour Soup Samples

Natural lycopene is the main pigment in ripe tomatoes and plays an important role in photosynthesis [37]. Natural lycopene is mostly located in the trans-structure [38], but its cis-structure is more bioactive and has a higher absorption rate [39]. The total lycopene content in the MZ sample increased to 63.73 ± 0.72μg/mL after five days of fermentation and was significantly higher than that in other samples (*p* < 0.05, Table 3). The total lycopene content in the MLQ sample decreased, but the content of cis-lycopene increased from 2.44 ± 0.03 μg/mL to 4.48 ± 0.01 μg/mL and the proportion of cis-lycopene in the MLQ sample (16.82 ± 0.36%) was the largest among all the samples. The total lycopene content or the proportion of cis-lycopene in MSQ samples did not change significantly (*p* ≥ 0.05) on day 1 or day 5 of fermentation. The total lycopene content and the proportion of cis-lycopene in MYQ samples also increased from 16.74 ± 0.26 μg/mL on day 1 to 30.15 ± 2.99μg/mL on day 5 and the proportion of cis-lycopene in MYQ samples also increased from 3.47 ± 0.78% to 7.11 ± 0.34%.

### 3.5. HS-GC-IMS Topographic Plots of Different Red Sour Soup Samples during Fermentation

**Figure 2:**
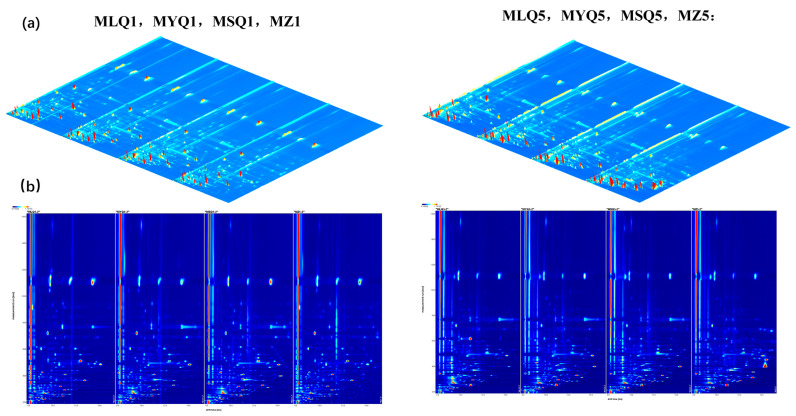
Topography of volatile flavor compounds in red sour soup prepared by different fermentation methods. (**a**) Three-dimensional spectra of the composition of volatile substances; (**b**) spectrum of comparative differences in the composition of volatile substances.

In this study, HS-GC-IMS was used to determine the flavor substances of different sour soup in various fermentation stages. Figure 2 shows the three-dimensional spectra and two-dimensional top views obtained by HS-GC-IMS. The composition of volatile substances in different samples could be compared visually. A white color indicated a lower intensity of volatile compounds and a red color indicated their higher intensity. On the first day of fermentation, MSQ had the highest signal intensity and natural fermentation had the lowest signal intensity (Figure 2b), while signal intensity showed no significant difference between MLQ and MYQ. On the fifth day of fermentation, the signal intensity of MZQ was the highest and the signal intensity of MSQ became the lowest.

### 3.6. Comparison of Fingerprint Profiles of Volatile Compounds in Different Red Sour Soup Samples

**Figure 3:**
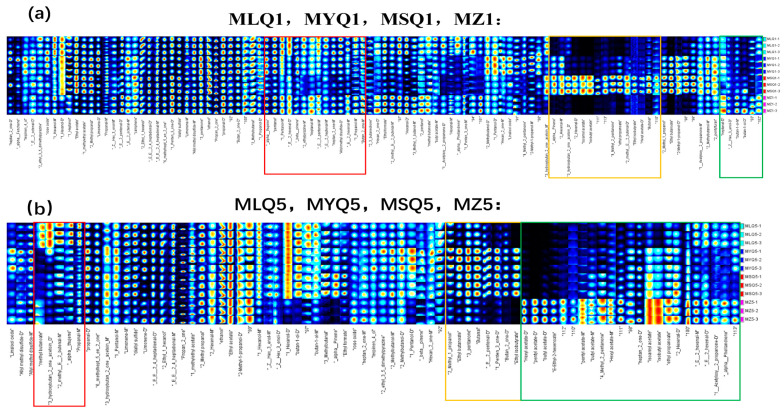
The fingerprints of different red sour soup samples; (**a**) indicates the first day of fermentation; (**b**) indicates the fifth day of fermentation.

To further identify the differences of volatile compounds among different sour soups, all the volatile compounds identified in the GC-IMS spectra were selected to generate volatile fingerprints using the Reporter plug-in (Figure 3). Each row of the fingerprint profile shows entire signal peaks of sour soup samples and each column of the fingerprint profile shows the signal intensities of the same compounds present in sour soup samples (Figure 3). In total, 119 compounds were qualitatively detected in fingerprint profiles (Figure 3). Among these volatiles, ninety-nine compounds were identified based on the GC-IMS database and NIST database (Table 4), including twenty-six alcohols, twenty-two aldehydes, eighteen ketones, seventeen esters, four ethers, three pyrazines, eight terpenoids, and one furan.

**Figure 4:**
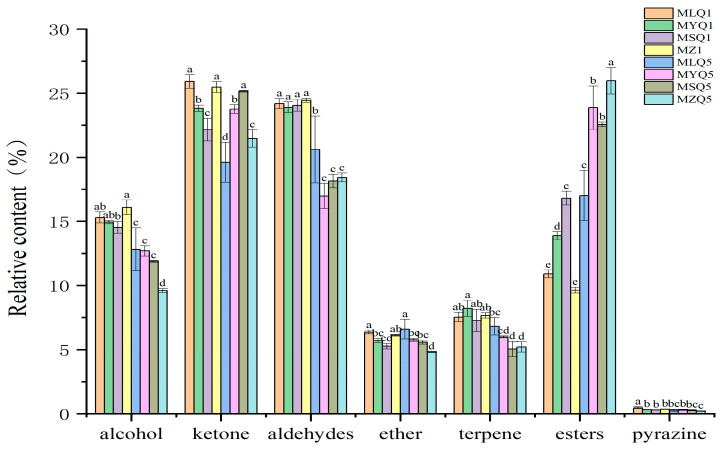
Relative content of volatile flavor components in different red sour soup samples. Different small letters indicate significant differences between samples of different red sour soups (*p* < 0.05).

Alcohols are the main volatile components in fresh fruits and contribute to a green and fresh odor [40]. In this study, alcohols were the most abundant compounds detected in red sour soup samples based on GC-IMS, including 26 compounds. The content of alcohols decreased in red sour soup samples on the fifth day compared to the first day (*p* < 0.05, Figure 4), probably due to the chemical reactions between alcohols and free fatty acids which formed esters during fermentation [41]. The content of esters increased in each sample (Figure 4). The content of some alcohols differed between samples. The content of (Z)-Hex-3-enol (D), butan-1-ol (M), and butan-1-ol (D) in MZ samples was higher than that in other samples on the first day of fermentation. The content of 1-Pentanol (M), (E)-2-pentenal (M), and 1-Propanol (M) in MLQ, MYQ, and MZ samples was significantly higher than that in MSQ samples on the first day of fermentation.

Aldehydes are mainly produced by unsaturated fatty acids through enzymatic and microbial oxidation and Strecker degradation [42] and often have pleasant, grassy, malty, and fruity flavors and aromas. Aldehydes have a low flavor threshold and a strong impact on the overall flavor of foods [43]. In this study, a total of seventeen aldehydes were detected by GC-IMS, including eight dimers. After five days of fermentation, the aldehyde content in each sample decreased compared to the first day (*p* < 0.05, Figure 4) because aldehydes were reduced into alcohols or oxidized into acids by microbial action [44] or as a result of lactic acid fermentation [45]. After one day of fermentation, MSQ samples contained much higher levels of 2-Hexenal (M), 2-methyl-(E)-2-butenal (D), and butanal, which endow red sour soup with clear, fruity, and nutty aromas, respectively, than the other three samples [45,46]. On the fifth day of fermentation, MZ samples contained higher levels of 2-Hexenal (M) and alpha-Phellandrene than the other three fermentation samples.

Ketones were the third major group of volatile flavor compounds determined in GC-IMS assays and eighteen ketones were found, including six dimers. Ketones are mainly produced by lipid oxidation, the Maillard reaction, and amino acid degradation [45] and endow fermented products with a unique fruitiness and aroma [47]. The reduction in the content of ketones in MLQ and MZ samples was ascribed to LAB. Silapeux et al. [48] also found that the inoculation of LAB in fermented fenugreek leaves could reduce the content of aldehydes and ketones and increase the content of esters. The content of 4-methyl-2-pentanone and 3-hydroxybutan-2-one-acetoin-D in the MSQ sample was higher than that in the other three samples in the first day of fermentation. On the fifth day of fermentation, the content of 3-pentanone, 1-Penten-3-one (D), and Butan-2-one (D), which endow the red sour soup with fruity, pungent, and garlicky flavors, in MLQ and MSQ samples was higher than that in the other two samples [49,50]. In contrast, the content of 4-methyl-2-pentanone, heptan-2-one (D), and 1-(Acetyloxy)-2-propanone (D) in the MZ sample was higher than that in the fortified fermentation samples. Heptan-2-one is produced by linoleic acid decomposition and has the odor of blue cheese [13].

In this study, a total of seventeen esters, including three dimers, were detected in the red sour soup samples based on GC-IMS. Esters are considered to be important flavor contributors in red sour soup [51]. Esters were not the first major class of compounds in this study and even small changes in ester content directly affected the sensory quality of the red sour soup due to the low flavor threshold of esters [52]. Different red sour soup samples showed an increased ester content on the fifth day of fermentation. Esters have pleasant, sweet, and fruity flavors [53] and are the main products in chili fermentation [54]. The ester content in MSQ samples on day 1 of fermentation was higher than that in other samples and the main esters included isoamyl acetate, isobutyl acetate, ethyl propanoate, and ethyl isobutyrate, which endow red sour soup with the flavors of various fruits such as banana and apple [55]. On the fifth day of fermentation, the content of some esters, mainly including Hexyl acetate (D), pentyl acetate (D), butyl acetate (D), and (E)-Ethyl-2-hexenoate, in MZ samples was significantly higher than that in the fortified fermentation samples. The MZ sample had a lot of miscellaneous bacteria and slow fermentation, so that the esters were only accumulated significantly on the fifth day.

Other compounds are explored below. Terpenes were mainly derived from raw materials and supporting materials, such as tomato, red pepper, and ginger, with floral and woody aromas [21]. The content of alpha-Fenchene in MSQ samples was significantly higher on the first day of fermentation. Ethers, with a low threshold and high contribution to the flavor of cooking wine, especially those containing benzene rings, mostly have strong and pleasant aromas and endow foods with special flavors [56]. Four ethers were detected in this study. Furan compounds are mainly generated by a Maillard reaction between amino acids and reducing sugars, and by a pyrolysis reaction between amino acids and thiamin, and have a strong meat flavor [57]. Only 2-pentylfuran was detected in this study.

### 3.7. Principal Component Analysis of Volatile Flavor Compounds in Different Red Sour Soup Samples

**Figure 5:**
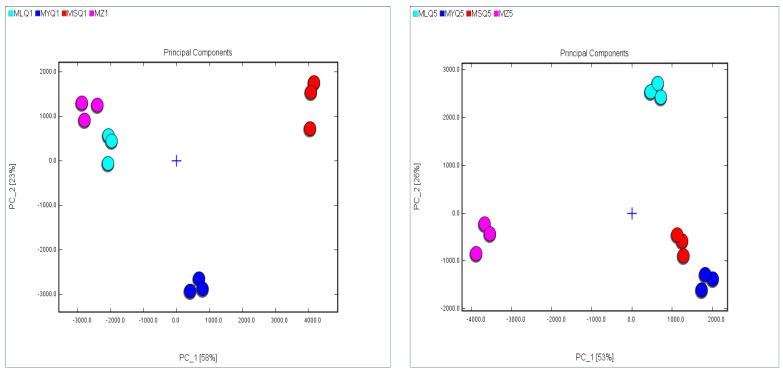
PCA based on the signal intensity obtained from different red sour soup samples.

PCA is a multivariate statistical analysis method in which several valid variables are selected through multivariate linear transformation. It is usually used to analyze the relationship between the observed variables. The PCA model can be chosen as a separation model when the cumulative contribution reaches 60% [58]. In this study, PCA was used to analyze the variation of identified VOC in red sour soup samples (Figure 5). The cumulative variance contribution of the first component PC1 (first principal component) (58%) and the second component PC2 (second principal component) (23%) was 81% on day 1 of fermentation. PC1 and PC2 showed high similarity between two samples, MLQ1 and MZ1. However, PC1 and PC2 showed significant differences between the remaining samples. On day 5 of fermentation, the cumulative variance contribution of the first component PC1 (first principal component) (58%) and the second component PC2 (second principal component) (23%) was 81%. PC1 and PC2 showed high similarity between two samples, MSQ5 and MYQ5. However, PC1 and PC2 showed significant differences between the remaining samples.

### 3.8. ROAV Analysis of Key Volatile Compounds in Different Red Sour Soup Samples

**Figure 6:**
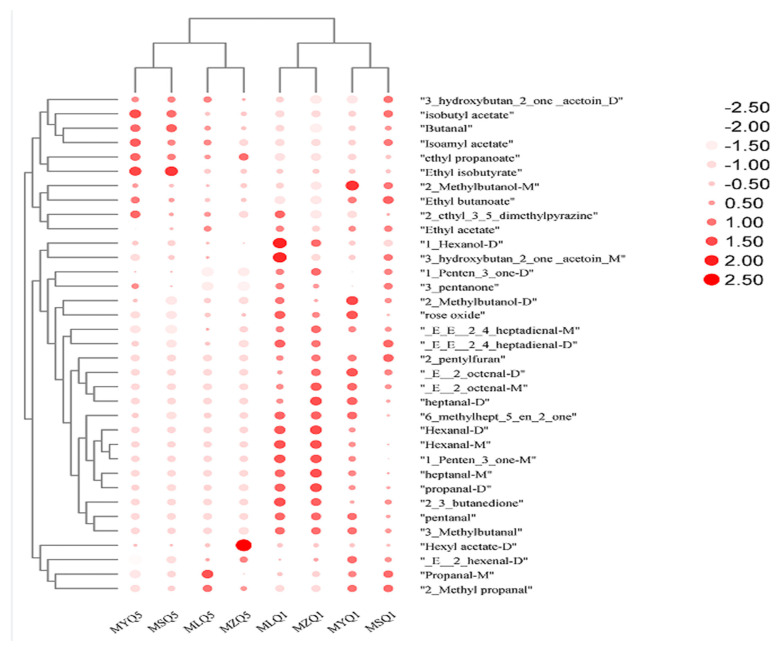
Heat accumulation map based on ROAV values of volatile flavor components in different red sour soup samples.

ROAV values indicate the contribution of key aromas to the overall flavor. ROAV is usually used to assess the impact of individual volatile compounds on the overall aroma. Compounds with ROAV ≥ 1 are considered key volatile aroma compounds with a high contribution to flavor [59]. On the first day of fermentation, 31, 32, 32, and 31 key volatile aroma compounds were found in MLQ, MYQ, MSQ, and MZ samples, respectively, and 28 aroma compounds were the same in three samples. On the fifth day of fermentation, 22, 22, 22, and 21 key volatile aroma components were found in MLQ, MYQ, MSQ, and MZ samples, respectively, and 21 aroma compounds were the same in three samples. On the fifth day of fermentation, hexyl acetate (D) was the key volatile compound (ROAV = 1.81) in the MZ sample and the ROAV of hexyl acetate (D) was less than 0.1 in the other samples (Table 5 and Figure 6). Hexyl acetate (D) endows red sour soup with apple and fruit flavors [60]. Propanal-M (ROAV = 9.74) and 2-Methyl propanal (ROAV = 139.26) contributed more to the aroma of MLQ samples than to that of other samples (Figure 6). 2-Methyl propanal has a fatty and mushroom-like odor. Isoamyl acetate, isobutyl acetate, ethyl isobutyrate, and butanal contributed significantly more to the aroma of MYQ and MSQ samples than to that of MLQ and MZ samples (Figure 6); isoamyl acetate and isobutyl acetate endow red sour soup with banana and fruity flavors and sweet and apple flavors, respectively [61]. Isoamyl acetate also shows a wide range of antibacterial activity against filamentous fungi [62]. Ethyl isobutyrate endows red sour soup with a strawberry flavor.

## 4. Conclusions

In this study, the effects of fortified fermentation with *L. buchneri* and/or *K. bulderi* on the basic physicochemical properties and volatile flavor substances of red sour soup were investigated. Compared to natural fermentation, fortified fermentation decreased the amino acid nitrogen content. Compared to the inoculation of *L. buchneri* and natural fermentation alone, the mixed fortified fermentation and the inoculation of *K. bulderi* could reduce the content of nitrite and increase the content of 3-pentanone and butyraldehyde. The inoculation of *L. buchneri* in fortified fermentation significantly increased the content of lactic acid. In addition, compared to fortified fermentation, natural fermentation had a higher content of lycopene and esters. Therefore, we will use omics technology (e.g., transcriptomics and metabolomics) to explore the results of fortified fermentation with the two strains. These results contribute to the understanding of the effect of fermentation agents on the fermentation of red sour soup and provide technical support for the industrial production of red sour soup.

## Figures and Tables

**Table 1:** The number of microbial colonies in red sour soup samples produced by different fermentation methods.

Sample	Number of Bacterial Colonies (10^6^ CFU/mL)	Total Number of LAB Colonies (10^6^ CFU/mL)	Total Number of Yeast Colonies (10^6^ CFU/mL)
MLQ1	1.28 ± 0.25 ^f^	3.60 ± 0.40 ^d^	1.43 ± 0.12 ^ef^
MLQ5	91.33 ± 1.53 ^b^	27.17 ± 0.31 ^b^	12.23 ± 0.31 ^c^
MYQ1	5.03 ± 0.25 ^e^	0.23 ± 0.02 ^f^	1.10 ± 0.10 ^ef^
MYQ5	4.90 ± 0.27 ^e^	4.93 ± 0.12 ^c^	64.67 ± 1.53 ^a^
MSQ1	13.80 ± 0.99 ^d^	2.90 ± 0.10 ^de^	2.00 ± 0.20 ^e^
MSQ5	149.33 ± 4.04 ^a^	285.90 ± 1.65 ^a^	48.67 ± 1.53 ^b^
MZ1	0.25 ± 0.01 ^f^	0.23 ± 0.02 ^e^	0.25 ± 0.01 ^f^
MZ5	74.33 ± 2.08 ^c^	2.40 ± 0.10 ^f^	7.50 ± 0.10 ^d^

Note: Different small letters indicate significant differences between samples of different red sour soups (*p* < 0.05).

**Table 2:** Changes in organic acids in different red sour soup samples.

Sample	Oxalic Acid (g/kg)	Tartaric Acid (g/kg)	Malic Acid (g/kg)	Lactic Acid (g/kg)	Acetic Acid (g/kg)	Citric Acid (g/kg)	Succinic Acid (g/kg)
MLQ1	0 ± 0 ^b^	0 ± 0 ^a^	7.79 ± 0.26 ^a^	2.91 ± 0.33 ^b^	0 ± 0 ^d^	3.77 ± 0.46 ^cd^	0 ± 0 ^c^
MLQ5	0 ± 0 ^b^	0 ± 0 ^a^	12.68 ± 8.43 ^a^	13.55 ± 11.77 ^a^	0.55 ± 0.31 ^cd^	3.59 ± 0.31 ^d^	0.54 ± 0.32 ^b^
MYQ1	0.02 ± 0.01 ^a^	0 ± 0 ^a^	8.31 ± 0.87 ^a^	4.26 ± 0.83 ^b^	0.40 ± 0.12 ^d^	3.45 ± 0.25 ^d^	0 ± 0 ^c^
MYQ5	0 ± 0 ^b^	0 ± 0 ^a^	12.09 ± 0.32 ^a^	5.33 ± 1.06 ^b^	1.42 ± 0.70 ^b^	3.30 ± 0.23 ^d^	0.23 ± 0.23 ^c^
MSQ1	0 ± 0 ^b^	0.17 ± 0.30 ^a^	7.98 ± 0.07 ^a^	3.37 ± 1.06 ^b^	1.22 ± 0.77 ^bc^	4.33 ± 0.06 ^bc^	0 ± 0 ^c^
MSQ5	0 ± 0 ^b^	0.04 ± 0.04 ^a^	11.83 ± 0.94 ^a^	0.22 ± 0.19 ^b^	3.41 ± 0.29 ^a^	0 ± 0 ^e^	3.96 ± 0.15 ^a^
MZ1	0 ± 0 ^b^	0 ± 0 ^a^	0.76 ± 0.30 ^a^	2.00 ± 0.36 ^b^	0.04 ± 0.07 ^d^	6.53 ± 0.73 ^a^	0 ± 0 ^c^
MZ5	0 ± 0 ^b^	0 ± 0 ^a^	13.43 ± 0.20 ^a^	1.94 ± 0.13 ^b^	0.26 ± 0.34 ^d^	4.69 ± 0.10 ^b^	0 ± 0 ^c^

Note: Different small letters indicate significant differences between samples of different red sour soups (*p* < 0.05).

**Table 3:** Changes in lycopene in different red sour soup samples.

Sample	Trans Lycopene ConCentration (ug/mL)	Cis-Lycopene (µg/mL)	Total Lycopene Content (ug/mL)	Cis-Proportion (%)
MLQ1	27.93 ± 0.03 ^d^	2.44 ± 0.03 ^d^	30.37 ± 0.06 ^c^	8.04 ± 0.09 ^c^
MLQ5	22.16 ± 0.54 ^e^	4.48 ± 0.01 ^b^	26.64 ± 0.53 ^d^	16.82 ± 0.36 ^a^
MYQ1	16.74 ± 0.26 ^f^	0.60 ± 0.13 ^e^	17.34 ± 0.13 ^e^	3.47 ± 0.78 ^e^
MYQ5	30.15 ± 2.99 ^c^	2.30 ± 0.11 ^d^	32.45 ± 3.10 ^c^	7.11 ± 0.34 ^d^
MSQ1	48.26 ± 0.26 ^b^	3.24 ± 0.01 ^c^	51.50 ± 0.27 ^b^	6.29 ± 0.01 ^d^
MSQ5	48.49 ± 0.48 ^b^	3.40 ± 0.12 ^c^	51.89 ± 0.36 ^b^	6.55 ± 0.28 ^d^
MZ1	13.93 ± 1.03 ^g^	0 ± 0 ^f^	13.93 ± 1.04 ^f^	0 ± 0 ^f^
MZ5	57.71 ± 0.63 ^a^	6.02 ± 0.64 ^a^	63.73 ± 0.72 ^a^	9.44 ± 0.94 ^b^

Note: Different small letters indicate significant differences between samples of different red sour soups (*p* < 0.05).

**Table 4:** GC-IMS global regional aggregation parameters obtained from different sour soup samples.

Count	Classification	Compound	GAS#	Formula	MW	RI	RT (sec)	DT (a.u)	Comment
1	alcohol	terpinen-4-ol	C562743	C_10_H_18_O	154.3	1599.4	1538.14	1.2293	
2		2-Ethyl-1-hexanol	C104767	C_8_H_18_O	130.2	1492.7	1114.76	1.4029	
3		Linalool	C78706	C_10_H_18_O	154.3	1514.6	1190.996	1.2202	
4		Linalool oxide	C60047178	C_10_H_18_O_2_	170.3	1417.4	888.137	1.267	
5		(Z)-Hex-3-enol	C928961	C_6_H_12_O	100.2	1401.2	845.938	1.2399	Monomer
6		(Z)-Hex-3-enol	C928961	C_6_H_12_O	100.2	1400.1	843.061	1.5122	Dimer
7		1-Hexanol	C111273	C_6_H_14_O	102.2	1368.5	766.334	1.3283	Monomer
8		1-Hexanol	C111273	C_6_H_14_O	102.2	1369.7	769.211	1.6428	Dimer
9		1-Pentanol	C71410	C_5_H_12_O	88.1	1261.2	560.649	1.2564	Monomer
10		1-Pentanol	C71410	C_5_H_12_O	88.1	1260.8	559.984	1.5146	Dimer
11		3-Methyl-1-butanol	C123513	C_5_H_12_O	88.1	1217.9	499.005	1.2482	Monomer
12		3-Methyl-1-butanol	C123513	C_5_H_12_O	88.1	1217.3	498.318	1.4957	Dimer
13		2-Methylbutanol	C137326	C_5_H_12_O	88.1	1169.5	439.224	1.2208	Monomer
14		2-Methylbutanol	C137326	C_5_H_12_O	88.1	1169.2	438.88	1.484	Dimer
15		(E)-2-pentenal	C1576870	C_5_H_8_O	84.1	1142.9	410.147	1.1059	Monomer
16		(E)-2-pentenal	C1576870	C_5_H_8_O	84.1	1142.7	409.898	1.3601	Dimer
17		2-pentanol	C6032297	C_5_H_12_O	88.1	1099.8	367.003	1.4533	
18		1-Propanol	C71238	C_3_H_8_O	60.1	1049.7	329.178	1.1128	Monomer
19		1-Propanol	C71238	C_3_H_8_O	60.1	1049.3	328.838	1.2534	Dimer
20		1-Heptanol	C111706	C_7_H_16_O	116.2	1000.6	296.173	1.3926	
21		ethanol	C64175	C_2_H_6_O	46.1	940.1	267.081	1.133	
22		2-Methyl-1-propanol	C78831	C_4_H_10_O	74.1	1094.7	362.546	1.3678	
23		butan-1-ol	C71363	C_4_H_10_O	74.1	1153.7	421.783	1.183	Monomer
24		butan-1-ol	C71363	C_4_H_10_O	74.1	1153	420.959	1.3819	Dimer
25		2-Methyl-1-propanol	C78831	C_4_H_10_O	74.1	1104	371.027	1.1733	Monomer
26		2-Methyl-1-propanol	C78831	C_4_H_10_O	74.1	1104.6	371.581	1.3654	Dimer
27	aldehydes	(E,E)-2,4-heptadienal	C4313035	C_7_H_10_O	110.2	1492.3	1113.399	1.1948	Monomer
28		(E,E)-2,4-heptadienal	C4313035	C_7_H_10_O	110.2	1491.5	1110.676	1.6334	Dimer
29		(E)-2-octenal	C2548870	C_8_H_14_O	126.2	1438.2	945.682	1.3364	Monomer
30		(E)-2-octenal	C2548870	C_8_H_14_O	126.2	1437.2	942.805	1.8237	Dimer
31		(E)-2-heptenal	C18829555	C_7_H_12_O	112.2	1330.3	682.894	1.258	Monomer
32		(E)-2-heptenal	C18829555	C_7_H_12_O	112.2	1330.7	683.853	1.6709	Dimer
33		(E)-2-hexenal	C6728263	C_6_H_10_O	98.1	1228	512.798	1.1814	Monomer
34		(E)-2-hexenal	C6728263	C_6_H_10_O	98.1	1227	511.469	1.5226	Dimer
35		heptanal	C111717	C_7_H_14_O	114.2	1194.6	468.771	1.3363	Monomer
36		heptanal	C111717	C_7_H_14_O	114.2	1194.6	468.771	1.6949	Dimer
37		Hexanal	C66251	C_6_H_12_O	100.2	1099	366.255	1.2669	Monomer
38		Hexanal	C66251	C_6_H_12_O	100.2	1097.7	365.008	1.5614	Dimer
39		2-methyl-(E)-2-butenal	C497030	C_5_H_8_O	84.1	1110.7	377.478	1.0925	Monomer
40		2-methyl-(E)-2-butenal	C497030	C_5_H_8_O	84.1	1110.7	377.478	1.3482	Dimer
41		pentanal	C110623	C_5_H_10_O	86.1	961.1	276.608	1.1845	
42		3-Methylbutanal	C590863	C_5_H_10_O	86.1	925.1	260.446	1.4038	
43		Propionaldehyde	C123386	C_3_H_6_O	58.1	826.1	220.689	1.0633	Monomer
44		propanal	C123386	C_3_H_6_O	58.1	826.4	220.792	1.1462	Dimer
45		2-Methyl propanal	C78842	C_4_H_8_O	72.1	805	213.004	1.0867	
46		2-Hexenal	C505577	C_6_H_10_O	98.1	1244.3	535.778	1.1648	Monomer
47		2-Hexenal	C505577	C_6_H_10_O	98.1	1244.3	535.778	1.5154	Dimer
48		Butanal	C123728	C_4_H_8_O	72.1	914.2	255.715	1.2901	
49	ketone	1-(Acetyloxy)-2-propanone	C592201	C_5_H_8_O_3_	116.1	1471.4	1045.331	1.1978	Dimer
50		1-(Acetyloxy)-2-propanone	C592201	C_5_H_8_O_3_	116.1	1471	1043.97	1.0943	Monomer
51		6-methylhept-5-en-2-one	C110930	C_8_H_14_O	126.2	1347.5	719.339	1.1766	
52		3-hydroxybutan-2-one (acetoin)	C513860	C_4_H_8_O_2_	88.1	1296.4	616.475	1.066	Monomer
53		3-hydroxybutan-2-one (acetoin)	C513860	C_4_H_8_O_2_	88.1	1296	615.81	1.3313	Dimer
54		heptan-2-one	C110430	C_7_H_14_O	114.2	1190.4	463.617	1.264	Monomer
55		heptan-2-one	C110430	C_7_H_14_O	114.2	1191.6	464.991	1.6335	Dimer
56		Hexan-2-one	C591786	C_6_H_12_O	100.2	1116.8	383.463	1.2028	Monomer
57		Hexan-2-one	C591786	C_6_H_12_O	100.2	1116.8	383.463	1.5048	Dimer
58		1-Penten-3-one	C1629589	C_5_H_8_O	84.1	1038.3	321.182	1.078	Monomer
59		1-Penten-3-one	C1629589	C_5_H_8_O	84.1	1037.6	320.672	1.3119	Dimer
60		3-pentanone	C96220	C_5_H_10_O	86.1	995.7	293.111	1.3564	
61		2,3-butanedione	C431038	C_4_H_6_O_2_	86.1	988.7	289.708	1.1727	
62		Butan-2-one	C78933	C_4_H_8_O	72.1	913.3	255.343	1.2472	Dimer
63		Butan-2-one	C78933	C_4_H_8_O	72.1	911.7	254.662	1.0669	Monomer
64		Propan-2-one	C67641	C_3_H_6_O	58.1	841.5	226.428	1.1136	
65		4-Methyl-2-pentanone	C108101	C_6_H_12_O	100.2	1024.7	311.955	1.18	
66		4-Methyl-2-pentanone	C108101	C_6_H_12_O	100.2	986.7	288.749	1.4801	
67	ethers	rose oxide	C16409431	C_10_H_18_O	154.3	1334.5	691.573	1.356	
68		Allyl methyl disulfide	C2179580	C_4_H_8_S_2_	120.2	1289.2	604.512	1.1118	Monomer
69		Allyl methyl disulfide	C2179580	C_4_H_8_S_2_	120.2	1289.2	604.512	1.4626	Dimer
70		diallyl sulfide	C592881	C_6_H_10_S	114.2	1158.6	427.106	1.1215	
71	pyrazine	2-ethyl-3,5-dimethylpyrazine	C13925070	C_8_H_12_N_2_	136.2	1435.1	937.051	1.2299	
72		2-ethylpyrazine	C13925003	C_6_H_8_N_2_	108.1	1297.8	619.133	1.1418	Monomer
73		2-ethylpyrazine	C13925003	C_6_H_8_N_2_	108.1	1297.4	618.468	1.519	Dimer
74	terpene	Limonene	C138863	C_10_H_16_	136.2	1204.8	481.826	1.2964	Monomer
75		Limonene	C138863	C_10_H_16_	136.2	1206.4	483.888	1.7248	Dimer
76		beta-pinene	C127913	C_10_H_16_	136.2	1125.3	391.942	1.211	
77		alpha-Fenchene	C471841	C_10_H_16_	136.2	1076	348.299	1.214	
78		camphene	C79925	C_10_H_16_	136.2	1059.7	336.329	1.2013	
79		alpha-thujene	C2867052	C_10_H_16_	136.2	1030.8	316.078	1.2165	
80		alpha-Phellandrene	C99832	C_10_H_16_	136.2	1212.7	492.134	1.6659	
81		alpha-Pinene	C80568	C_10_H_16_	136.2	1025.2	312.26	1.2924	
82	esters	methyl butanoate	C623427	C_5_H_10_O_2_	102.1	1001.1	296.513	1.4267	
83		Ethyl acetate	C141786	C_4_H_8_O_2_	88.1	895.5	247.857	1.3356	
84		Ethyl formate	C109944	C_3_H_6_O_2_	74.1	866.6	236.163	1.0581	
85		1-methylethyl acetate	C108214	C_5_H_10_O_2_	102.1	851.9	230.424	1.1554	
86		Ethyl butanoate	C105544	C_6_H_12_O_2_	116.2	1047.8	327.82	1.5594	
87		Isoamyl acetate	C123922	C_7_H_14_O_2_	130.2	1132.5	399.316	1.7516	
88		isobutyl acetate	C110190	C_6_H_12_O_2_	116.2	1026.2	312.969	1.6185	
89		ethyl propanoate	C105373	C_5_H_10_O_2_	102.1	968.4	280.009	1.4559	
90		Ethyl isobutyrate	C97621	C_6_H_12_O_2_	116.2	979.2	285.153	1.5619	
91		(E)-Ethyl-2-hexenoate	C27829727	C_8_H_14_O_2_	142.2	1328.3	678.772	1.8187	
92		Hexyl acetate	C142927	C_8_H_16_O_2_	144.2	1284.6	597.075	1.3905	Monomer
93		Hexyl acetat	C142927	C_8_H_16_O_2_	144.2	1282.7	593.932	1.9018	Dimer
94		pentyl acetate	C628637	C_7_H_14_O_2_	130.2	1182.1	453.79	1.3124	Monomer
95		pentyl acetate	C628637	C_7_H_14_O_2_	130.2	1182.6	454.418	1.7653	Dimer
96		butyl acetate	C123864	C_6_H_12_O_2_	116.2	1086.3	356.11	1.2392	Monomer
97		butyl acetate	C123864	C_6_H_12_O_2_	116.2	1084.8	354.932	1.6202	Dimer
98		methyl acetate	C79209	C_3_H_6_O_2_	74.1	856.3	232.13	1.1917	
99		2-pentylfuran	C3777693	C_9_H_14_O	138.2	1238.9	528.084	1.2546	

Note: MW means the molecule weight of the volatiles; RT means the retention time of the volatiles on GC-IMS; RI means the retention index of the volatiles on capillary column; DT means the drift time of the volatiles on GC-IMS.

**Table 5:** The ROAV values of volatile flavor compounds in different red sour soup samples.

Compound	T	ROAV							
		MLQ1	MYQ1	MSQ1	MZQ1	MLQ5	MYQ5	MSQ5	MZQ5
“1_Hexanol-D”	0.0056	6.05	1.74	1.15	4.60	2.31	1.84	1.45	2.55
“2_Methylbutanol-M”	0.0159	1.64	2.24	2.06	1.51	1.77	1.94	1.74	1.69
“2_Methylbutanol-D”	0.0159	1.20	1.33	1.11	1.02	0.79	0.86	0.61	0.69
“_E_E__2_4_heptadienal-M”	0.0154	7.26	7.20	7.07	7.48	6.78	5.62	5.46	5.95
“_E_E__2_4_heptadienal-D”	0.0154	10.10	7.97	10.02	9.72	7.22	6.70	5.72	5.95
“_E__2_octenal-M”	0.0002	110.25	140.62	103.08	153.23	-	-	-	-
“_E__2_octenal-D”	0.0002	22.22	37.73	28.06	32.79	-	-	-	-
“_E__2_hexenal-D”	0.0885	1.00	1.44	1.28	1.18	1.17	0.37	0.67	1.35
“heptanal-M”	0.005	3.46	2.78	1.81	3.93	-	-	-	-
“heptanal-D”	0.005	1.16	1.60	0.92	1.80	-	-	-	-
“Hexanal-M”	0.005	11.30	7.36	3.60	11.43	-	-	-	-
“Hexanal-D”	0.005	43.47	28.45	17.39	46.86	-	-	-	-
“pentanal”	0.012	20.30	19.29	12.35	20.14	-	-	-	-
“3_Methylbutanal”	0.0004	303.30	294.88	227.69	301.52	39.88	36.82	47.16	8.46
“Propanal-M”	0.0151	6.37	8.65	9.15	5.70	9.74	4.71	5.55	7.29
“propanal-D”	0.0151	21.26	18.99	17.89	22.03	14.18	14.22	13.57	13.29
“2_Methyl propanal”	0.0015	111.27	139.20	140.15	111.08	139.26	109.46	115.34	132.45
“Butanal”	0.002	24.91	31.89	70.40	5.21	69.98	88.22	91.91	40.68
“3_hydroxybutan_2_one _acetoin_M”	0.014	3.22	1.35	2.48	1.09	1.96	1.04	1.38	1.82
“3_hydroxybutan_2_one _acetoin_D”	0.014	1.97	0.71	5.84	0.53	5.42	5.17	5.38	4.15
“6_methylhept_5_en_2_one”	0.068	3.05	3.01	2.23	3.06	1.95	2.01	1.71	1.85
“1_Penten_3_one-M”	0.023	1.81	1.27	0.63	1.83	-	-	-	-
“1_Penten_3_one-D”	0.023	13.03	9.52	12.95	14.36	1.97	9.79	9.85	2.78
“3_pentanone”	0.06	5.34	4.96	5.36	5.17	4.24	5.26	4.98	4.15
“2_3_butanedione”	0.01	7.16	3.70	4.64	6.56	-	-	-	-
“2_ethyl_3_5_dimethylpyrazine”	0.00004	441.94	277.99	373.56	254.29	398.14	441.34	382.28	283.28
“rose oxide”	0.0001	299.27	308.26	179.40	257.39	158.34	114.56	93.98	170.99
“Ethyl acetate”	0.005	67.30	65.94	67.55	47.50	66.07	57.72	53.98	31.63
“Ethyl butanoate”	0.0009	13.99	265.60	304.47	3.81	112.85	270.51	216.93	109.99
“Isoamyl acetate”	0.00015	7.45	99.07	581.83	10.18	525.90	684.66	535.55	38.50
“isobutyl acetate”	0.073	0.02	0.16	1.28	0.02	0.38	1.54	1.31	0.32
“ethyl propanoate”	0.01	0.18	2.72	5.92	0.15	13.54	18.75	16.29	18.06
“Hexyl acetate-D”	0.115	0.02	0.04	0.13	0.02	0.05	0.14	0.18	1.81
“Ethyl isobutyrate”	0.0009	1.50	2.43	10.77	1.48	2.72	122.84	133.21	3.35
“2_pentylfuran”	0.0058	3.14	3.61	4.15	3.67	-	-	-	-

Note: “-”means not detected or the detection limit was too low.

## Data Availability

Data will be made available on request.
